# Localised Peritonitis1Read at the twenty-eighth annual meeting of the Medical Association of Central New York, at Syracuse, October 15, 1895.

**Published:** 1896-02

**Authors:** C. O. Baker

**Affiliations:** Auburn. 65 East Genesee Street


### LOCALISED PERITONITIS.[1]

By C. O. BAKER, M. D., Auburn.

I WISH simply to detail a few cases of peritonitis which have clearly had a local origin :

CASE I.—In August, 1892, I saw a woman, twenty years old, who twenty-four hours before my visit was taken violently ill with pain in the abdomen, of a diffuse character, accompanied with great swelling and tenderness. In short, all the symptoms of peritonitis were present in a severe form, demanding immediate relief.

As the cause of the condition was not clear, celiotomy was decided upon and search made for the lesions, which were found to be under the gastro-splenic omentum, where there was found a circumscribed collection of pus, about one drachm, walled in by the matting together of the surrounding tissues. The pus was washed away, adhesions broken and the wound closed without drainage. Recovery was uneventful and rapid.

. This woman gave a history of a misstep while walking on the sidewalk, causing sudden and severe pain in the region of the spleen.

CASE II.—May 30, 1893, I saw Mrs. L., aged 32. She gave a history of ten days' sickness, beginning with localised pain in the region of the spleen, rapidly extending over the abdomen. At my first visit the abdomen was so swollen as to make it quite impossible to determine the condition of its contents. Section was made. Exploration revealed a large amount of free fluid and the spleen displaced downward and enlarged to seven or eight times its normal size, the gastro-splenic omentum constricted by bands of inflammatory origin, interfering with the circulation of that organ. The spleen was punctured and allowed to bleed freely. The inflammatory bands were destroyed, the wound

---

1. Read at the twenty-eighth annual meeting of the Medical Association of Central New York, at Syracuse, October 15, 1895.

closed with drainage, which was left until the spleen had recovered its vitality (fifty-two hours). No cause is known in this case for the local peritonitis causing the trouble with the spleen. Patient made a perfect recovery.

CASE III.—In June, 1893, I made an exploration of the abdomen of a man, an inmate of Auburn prison, who had a large, hard growth in the region of the spleen, which followed an attack of localised peritonitis, occurring three months previous to my examination. I found the spleen spherical in form and about six and one-half inches in diameter, very hard and the color of dark venous blood. The vessels in the gastro-splenic omentum were firmly constricted by inflammatory bands in such a manner as to greatly retard the return circulation. These bands were broken and the tumor gradually reduced in size. The supposed cause was lifting heavy weights while working in the foundry. The man was discharged from Auburn prison and lost from observation.

CASE IV.—In July, 1895, I saw Mrs. M., aged 45. She gave the following history—namely, eighteen months previous to my visit she suffered severely from the passage of a gall-stone, followed by localised peritonitis, from which condition she had never recovered. At my first visit I found her extremely emaciated, troubled with constant vomiting, complete paraplegia, the stomach displaced to the level of the umbilicus by a firm inelastic growth. Section was made and exploration revealed enlarged gall-bladder, with walls one-fourth of an inch in thickness, and containing about one gallon of brownish yellow fluid. The contents of the sac was drawn off. An examination of the gall-duct showed it to be firmly constricted, completely closed. The adhesions constricting the gall-duct were broken up and the duct dilated, establishing communication with the bowel, drainage-tube introduced and wound closed. At the end of the first week the biliary secretions were found mixed with the feces. The drainage opening was allowed to close and the patient took and retained food.

65 EAST GENESEE STREET.

---

IN THE Tuilleries Garden stands a circular building, devoted to the history of France for 100 years, shown by the patriotic figures of the great characters of the country during that period of time. One of the most interesting sights in Paris during the last great exhibition, was this striking pictorial view of what France had done in war, science and art for a century. Standing out on the canvas, in a most life-like manner, from a great circular panorama of kings, emperors, generals, statesmen and artists, is the figure of Pasteur. It is, indeed, a delight to see a great medical scientist thus honored; although it may be truly said that France honors itself in honoring Pasteur.—*Post-Graduate.*

